# Assessing the prognostic value of early oculomotor abnormalities in Huntington’s disease

**DOI:** 10.3389/fneur.2026.1844433

**Published:** 2026-06-15

**Authors:** Ahmad Kaddoura, Solveig E. J. Dalbro, Marleen R. van Walsem, Lasse Pihlstrøm

**Affiliations:** 1 Department of Neurology, Oslo University Hospital, Oslo, Norway; 2 Institute of Clinical Medicine, Faculty of Medicine, University of Oslo, Oslo, Norway

**Keywords:** biomarker, Enroll-HD, eye movements, Huntington's disease, prognosis

## Abstract

**Background:**

Abnormalities of eye movements occur early in the disease course of Huntington’s disease, often preceding clinical motor diagnosis. The increasing availability of digital eye tracking tools indicate a potential role for eye movements as biomarkers of prognosis and progression in Huntington’s disease, yet the optimal set of oculomotor tasks to measure is currently unknown.

**Methods:**

We explored the clinical progression of the six oculomotor items of the Unified Huntington’s Disease Rating Scale in data from the Enroll-HD (*N* = 4,775) and PREDICT-HD (*N* = 969) observational studies and their association with time to clinical motor diagnosis and functional decline in time-dependent survival models.

**Results:**

Vertical eye movements were affected in larger proportions of participants before motor diagnosis than horizontal eye movements. Stepwise Cox proportional hazard regression analyses identified saccade velocity, vertical smooth pursuit, and horizontal saccade initiation as independently significant predictors of progression to motor manifest disease and functional decline.

**Conclusion:**

Oculomotor progression follows a distinct pattern in early Huntington’s disease, which should be kept in mind when developing digital eye tracking paradigms to be used as biomarkers in future observational studies and clinical trials.

## Introduction

Huntington’s disease (HD) is an autosomal dominant neurodegenerative disorder caused by an expanded CAG-repeat in exon 1 of *HTT*, encoding the protein huntingtin (1). HD causes progressive motor, cognitive and neuropsychiatric symptoms, typically beginning in adulthood. In common practice, a clinical diagnosis requires the presence of motor manifestations that are unequivocal signs of HD. However, this milestone is arbitrarily defined, and usually preceded by more subtle, non-specific motor signs. An improved understanding of the clinical relevance of specific early motor signs in predicting progression to manifest HD could have important implications for clinical trial design.

Oculomotor deficits emerge in presymptomatic gene carriers well before the appearance of unequivocal clinical signs (2–5). Quantitative eye-tracking studies reveal impairment of saccades, particularly during cognitively demanding anti-saccade and memory-guided tasks (6–9). Powerful new digital tools are currently revolutionizing clinical oculomotor assessment and may provide valuable new biomarkers for tracking disease progression in HD (9–11). Anticipating this development, it is warranted to explore in further detail the predictive value of the oculomotor items of the Unified HD Rating Scale-Total Motor Score (UHDRS-TMS) in available large longitudinal datasets.

Predicting phenotypic progression to clinical motor onset in premanifest HD in order to underpin clinical trial design has been a key aim of several ambitious HD projects (12, 13). Some previous studies have explored advanced statistical approaches to model individual risk of phenoconversion, including joint longitudinal survival models based on various clinical and paraclinical measures (14, 15). However, the pattern and prognostic significance of oculomotor progression have been specifically addressed primarily in studies of smaller sample size (16). A recent systematic review identified 20 published digital eye-tracking studies in HD, some including premanifest carriers, which have used a variety of different paradigms in small samples (9).

In this study, we took advantage of available clinical data from Enroll-HD and PREDICT-HD to explore the progression of UHDRS-TMS oculomotor items in premanifest HD. We investigated the power of time-dependent Cox regression models based on oculomotor findings to predict motor onset and onset of functional decline in HD, highlighting a biomarker potential that holds promise for future implementation of digital eye tracking technologies in HD research.

## Methods

We analyzed data from Enroll-HD (PDS6-R2) and PREDICT-HD (Release 7), which are large, longitudinal observational studies of HD. Details on design and study protocols have been published elsewhere (17, 18). Both studies have been approved by relevant ethical committees or review boards and all participation has been based on written, informed consent.

Primary analyses were performed in the Enroll-HD dataset. We defined a diagnosis of motor manifest HD as corresponding to Diagnostic Confidence Level (DCL) = 4 (19), a definition widely used both in trials and clinical practice to date. As an alternative outcome of interest, we repeated analyses using the UHDRS total functional capacity (TFC) < 13 to define transition from HD stage 2 to stage 3 according to the recently proposed integrated staging system for HD (HD-ISS) (20).

As our focus of interest was on oculomotor abnormalities preceding diagnosis, we included only participants with an expanded CAG repeat in the *HTT* gene (≥ = 36 repeats) and recorded data from at least two visits in the premanifest stage. The participant selection process is illustrated in Supplementary Figure 1. For external validation, analogous inclusion criteria were applied to the PREDICT-HD dataset. For analyses based on functional decline, we included participants with at least two visits with TFC = 13, resulting in a slightly larger sample size for this analysis (Table 1).

**Table 1:** Baseline characteristics of study participants.

Demographic variable	Enroll-HD (DCL = 4)	Enroll-HD (TFC < 13)	PREDICT-HD (DCL = 4)
Included participants, *N*	4,413	4,775	969
Participants reaching study endpoint, *n*	591	965	206
Age at baseline	39.4 (11.9)	40.3 (12.2)	39.6 (10.4)
CAG	42.2 (2.7)	42.5 (2.8)	42.4 (2.6)
Female sex, *n* (%)	2,661 (60.3%)	2,744 (57.5%)	621 (64.1%)
Median follow-up time, years (IQR)	3.51 (2.12–5.21)	3.25 (2.04–5.04)	4.1 (2.4–7.8)

Values are presented as mean (SD) unless otherwise specified. Follow-up time is presented as median (IQR). Endpoint defined as transition to DCL = 4 in the DCL cohorts and decline to TFC <13 in the TFC cohort. DCL, diagnostic confidence level; TFC, total functional capacity; IQR, interquartile range; SD, standard deviation.

The UHDRS-TMS includes 15 motor items in total, of which six assess oculomotor function, namely pursuit, saccade initiation and saccade velocity, all three in the horizontal and vertical direction. These items are scored on an ordinal scale from 0 (normal) to 4 (severely impaired) by trained examiners.

All analyses were performed in R (version 2024.12.1). We first explored the frequency and timing of oculomotor signs preceding DCL = 4 in the Enroll-HD dataset. The proportions of participants with score > 0 for each of the six oculomotor items at different timepoints up to motor diagnosis were visualized using Kaplan–Meier survival plots. The distribution of oculomotor scores from 0 to 4 for each item at each timepoint was displayed using barplots.

Next, we explored the association between oculomotor scores and hazard of subsequent motor diagnosis using time-dependent Cox proportional hazards models, which were fitted using the *survival* R package. Time since first premanifest visit was used as the underlying time scale, and data were structured in start–stop format, allowing covariates to be updated at each visit. Firstly, oculomotor variables were coded as binary time-dependent covariates (normal vs. abnormal) and updated at each visit. This represents a simplification, however, as scores higher than 1 are not given more weight. All models were adjusted for sex and the study-specific version of the CAG-age product (CAP score), a validated measure of expected disease burden in HD which is defined using slightly different algorithms in Enroll-HD (21) and PREDICT-HD (22). We chose to use CAP score rather than the formula published by Langbehn et al. predicting age at diagnosis defined as DCL = 4 based on CAG-repeats and age, as our analyses also included another event of interest (TFC < 13). CAP score was defined as a time-dependent variable, updated at each visit.

Forward stepwise selection was applied to the Enroll-HD dataset to identify independent oculomotor predictors, starting with the strongest univariable predictor and adding variables sequentially if they contributed significantly to the model (*p* < 0.05). Selection terminated when no additional variables improved model fit. We validated the performance of the final model in the independent PREDICT-HD dataset. Model discrimination was assessed using Harrell’s concordance index (C-index) with 95% confidence intervals. Bootstrap-corrected C-index values were calculated for the final models, and person-years at risk were reported. The proportional hazards assumption was evaluated using global Schoenfeld tests. The same Cox regression analyses were repeated using TFC < 13 as the event of interest.

Next, we explored the possibility of using the full scale of abnormal UHDRS oculomotor scores, rather than a binary distinction between normal and abnormal. Cox regression analyses were repeated treating oculomotor UHDRS items as ordinal quantitative variables rather than binary variables. Different coding strategies, including binary (0 vs. ≥ 1), linear (0–4), and grouped categorical models (0/1 / ≥2), were compared using Akaike and Bayesian information criteria (AIC/BIC). We also assessed pairwise hazard ratios across oculomotor scores 0, 1 and ≥2.

## Results

### Demographics

We included data from a total of 4,413 participants in Enroll-HD and 969 participants in PREDICT-HD for the analyses assessing progression to DCL = 4. In the analysis using TFC < 13 as event, we included 4,775 participants from Enroll-HD. Baseline characteristics of these cohorts are shown in Table 1. Age, CAG repeat length and sex distributions were broadly similar across the included datasets.

### Descriptive analyses of oculomotor abnormalities

**Figure 1:**
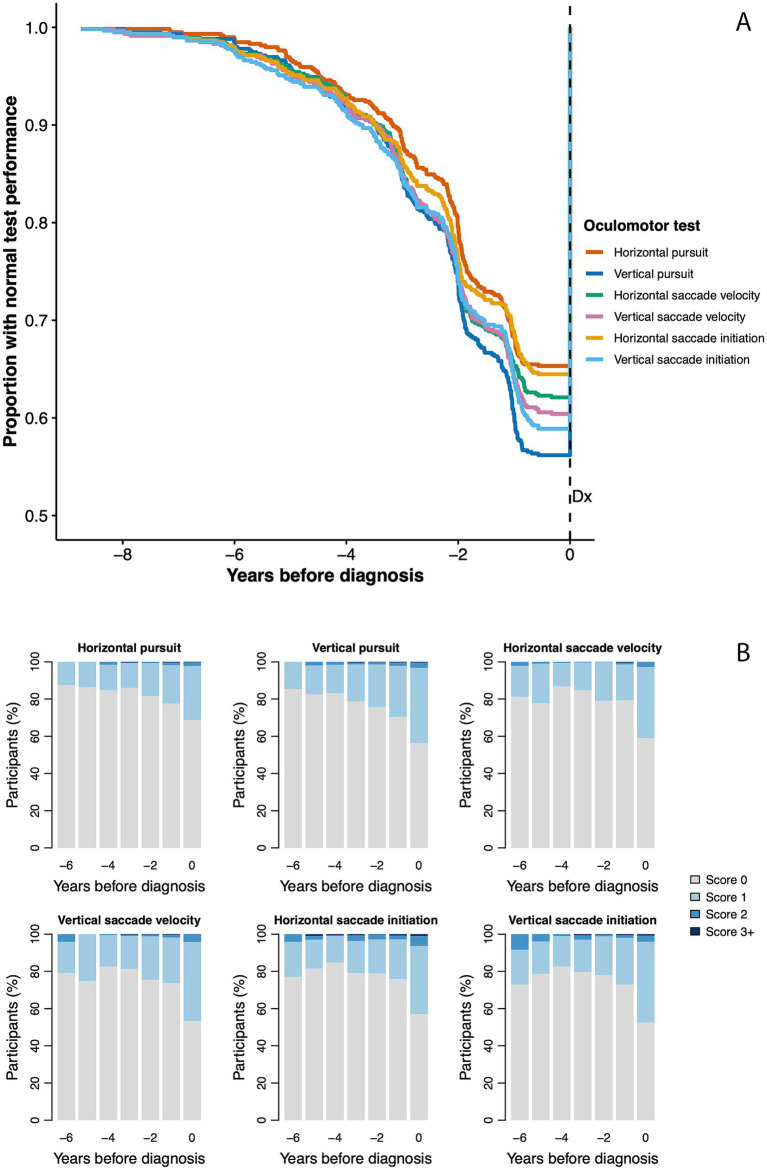
Oculomotor abnormalities preceding motor diagnosis in Enroll-HD data. **(A)** Kaplan–Meier curves represent the proportion of participants remaining with normal performance for each oculomotor item as a function of years before diagnosis. Year 0 denotes the first visit defined as motor manifest. Only participants who transitioned to manifest Huntington’s disease are included. Note that in order to visualize the differences across items, the *Y* axis starts at 50%. **(B)** Distribution of oculomotor scores by years before diagnosis. Bar plots show the proportion of participants with scores 0, 1, 2, and ≥3 for each oculomotor item across time before the first motor manifest visit. Only participants who transitioned to manifest Huntington’s disease are included and the number of included participants will be highest closest to diagnosis. Values are presented as percentages for each time point.

Figure 1A shows Kaplan–Meier curves visualizing the proportion of participants remaining with normal clinical ratings for each of the oculomotor items of the UHDRS-TMS in the years leading up to clinical diagnosis. Barplots of each item showing the distribution of clinical scores at each time point are shown in Figure 1B. Higher scores (≥2) were rare at all time points, including at the first visit defined as motor manifest. All oculomotor measures showed a trend of gradual decline in the proportion of participants with normal performance as diagnosis approached. The most smooth and consistent decline in proportion of normal assessments over time was seen for vertical pursuit, which also was the oculomotor item affected in the largest proportion of individuals at diagnosis. The other vertical eye movements were also more commonly affected than the horizontal movements. For all items, the most pronounced shift in proportion with abnormal score was seen from the last premanifest to the first visit categorized as motor manifest (DCL = 4).

### Cox proportional hazards models of time to clinical diagnosis

In the Enroll-HD cohort, all six oculomotor items were significantly associated with increased risk of transition to manifest Huntington’s disease when modeled individually as time-dependent binary predictors adjusted for CAP score and sex (Table 2). Hazard ratios ranged from approximately 1.61 to 2.07, with vertical saccade velocity showing the strongest association (HR 2.07, 95% CI 1.71–2.51, *p* < 0.001). Model discrimination was moderate across single-item models, with concordance indices ranging from 0.73 to 0.75. The proportional hazards assumption was satisfied for all predictors, with no evidence of global violation.

**Table 2:** Binary time-dependent Cox regression analyses in the DCL = 4 cohorts.

Oculomotor item normal vs abnormal	Enroll-HD, single-item HR (95% CI) Schoenfeld P	Enroll-HD, final model HR (95% CI)	PREDICT-HD, final model HR (95% CI)
Vertical smooth pursuit	1.83 (1.51–2.21), <0.001 PH = 0.710	1.43 (1.16–1.77), <0.001	1.54 (1.13–2.08), 0.006
Horizontal smooth pursuit	1.61 (1.30–1.99), <0.001 PH = 0.950	—	—
Vertical saccade velocity	2.07 (1.71–2.51), <0.001 PH = 0.740	1.60 (1.26–2.02), <0.001	1.55 (1.12–2.15), 0.009
Horizontal saccade velocity	1.89 (1.53–2.33), <0.001 PH = 1.000	—	—
Vertical saccade initiation	1.86 (1.52–2.27), <0.001 PH = 0.960	—	—
Horizontal saccade initiation	1.88 (1.53–2.31), <0.001 PH = 0.880	1.30 (1.02–1.66), 0.034	2.45 (1.77–3.38), <0.001
Model performance			
C-index (95% CI)	—	0.754 (0.734–0.774)	0.817 (0.787–0.848)
Bootstrap-corrected C-index	—	0.752	0.819
Person-years at risk	—	16,751	4,900
Global Schoenfeld *p*-value	—	0.83	0.03

Values are hazard ratios (HR) with 95% confidence intervals and p-values. PH indicates *p*-value from Schoenfeld residual test of the proportional hazard’s assumption. Single-item and multivariable Cox models were adjusted for CAP score and sex.

Forward stepwise selection identified three oculomotor items as independently significant predictors of transition to manifest HD when defined as binary variables: vertical saccade velocity, vertical smooth pursuit, and horizontal saccade initiation (Table 2). CAP score and male sex were also independently associated with increased hazard (data not shown). The final model demonstrated moderate discriminative ability (C-index = 0.754), with minimal optimism after bootstrap correction (bootstrap-corrected C-index = 0.752). The proportional hazards assumption was satisfied (global Schoenfeld test *p* = 0.83).

The three-item model developed on Enroll-HD data was applied to the PREDICT-HD cohort, where all three oculomotor predictors remained independently associated with transition after adjustment for CAP score and sex, and model discrimination was good (C-index = 0.82) (Table 2). In contrast to the Enroll-HD model, horizontal saccade initiation showed the strongest association in the validation cohort, whereas vertical saccade velocity and vertical smooth pursuit demonstrated quite similar effect sizes. The proportional hazards assumption was satisfied for the individual oculomotor predictors, although the global Schoenfeld test indicated a modest deviation from proportional hazards in the overall model (global *p* = 0.03), primarily driven by CAP score (*p* = 0.017).

### Cox proportional hazards models of time to stage 3 HD

Using a functional definition of stage 3 onset based on transition to TFC < 13, all six oculomotor abnormalities were significantly associated with increased risk of functional decline in single-item time-dependent Cox models adjusted for CAP score and sex (Supplementary Table 1). Hazard ratios ranged from approximately 1.63 to 2.15, with the strongest associations observed for saccadic measures. In the multivariable model derived using forward stepwise selection, the same three oculomotor items that were identified in the DCL = 4 analysis also emerged as independently associated with functional decline: vertical saccade velocity, vertical smooth pursuit, and horizontal saccade initiation (Supplementary Table 1). The final model demonstrated moderate discriminative ability (C-index = 0.730), with minimal optimism after bootstrap correction (bootstrap-corrected C-index = 0.727). The proportional hazards assumption was satisfied (global Schoenfeld test *p* = 0.34).

### Quantitative cox analyses and assessment of linearity

To further explore whether increasing oculomotor severity was associated with progressively increasing hazard, supplementary time-dependent Cox analyses were performed using ordered categorical or linear oculomotor UHDRS scores rather than binary abnormal/normal definitions. Comparison of different coding strategies using AIC/BIC generally favored binary coding over other coding startegies in the DCL = 4 cohort, whereas findings in the TFC < 13 cohort were more mixed (Supplementary Table 2). Exploratory pairwise analysis of score groups consistently yielded hazard ratios above 1 for scores of 1 versus 0. In contrast, when scores of ≥2 were compared to 1, we observed variable hazard ratios, many below one, indicating a lack of linear risk increase with increasing oculomotor item scores (Supplementary Table 3).

Despite these disencouraging results, we performed exploratory Cox regression analyses using oculomotor items as linear variables. The results are shown in Supplementary Tables S1, S4. The final models yielded similar or lower C-index compared with their binary counterpart, indicating again that quantitative coding did not improve model performance.

## Discussion

Oculomotor abnormalities are core clinical features of HD and affected early in the disease course of many patients. Despite causing limited subjective complaints and functional decline in the earliest manifest phase, eye movements may provide a window into the neurodegeneration and hold promise as biomarkers for early diagnosis and disease progression, especially with the increasing availability of objective quantitative digital eye tracking tools. In this study, we explored the progression of UHDRS-TMS oculomotor items from the premanifest stage in Enroll-HD and PREDICT-HD data, evaluating the performance of models based on clinical eye movement assessments in predicting conversion to clinically manifest HD (DCL = 4) or stage 3 HD (TFC < 13).

Vertical smooth pursuit was the oculomotor item affected in the largest proportion of individuals at diagnosis and showed the most consistent gradual increase in proportion affected over the years leading up to DCL = 4. This is in line with previous reports (5, 23) Vertical gaze centers in the rostral midbrain appear more vulnerable to neurodegeneration and metabolic stress than the pontine horizontal centers, as suggested by preferential vertical eye movement deterioration in healthy ageing (24, 25). Vertical eye movements are often neglected in digital eye tracking studies due to their technical complexity, but our results imply that they should be prioritized as the earliest and most consistent signals of impending phenoconversion. A model incorporating smooth pursuit, saccade latency, and saccade velocity potentially captures dysfunction across multiple interconnected oculomotor substrates. Smooth pursuit reflects cortical and cerebellar integrity, saccade latency indexes a network that begins in the frontal eye fields, passes through the basal ganglia and colliculus superior and releases the brainstem burst generator while decreased saccade velocity is mainly a result of brainstem dysfunction (23).

**Figure 2:**
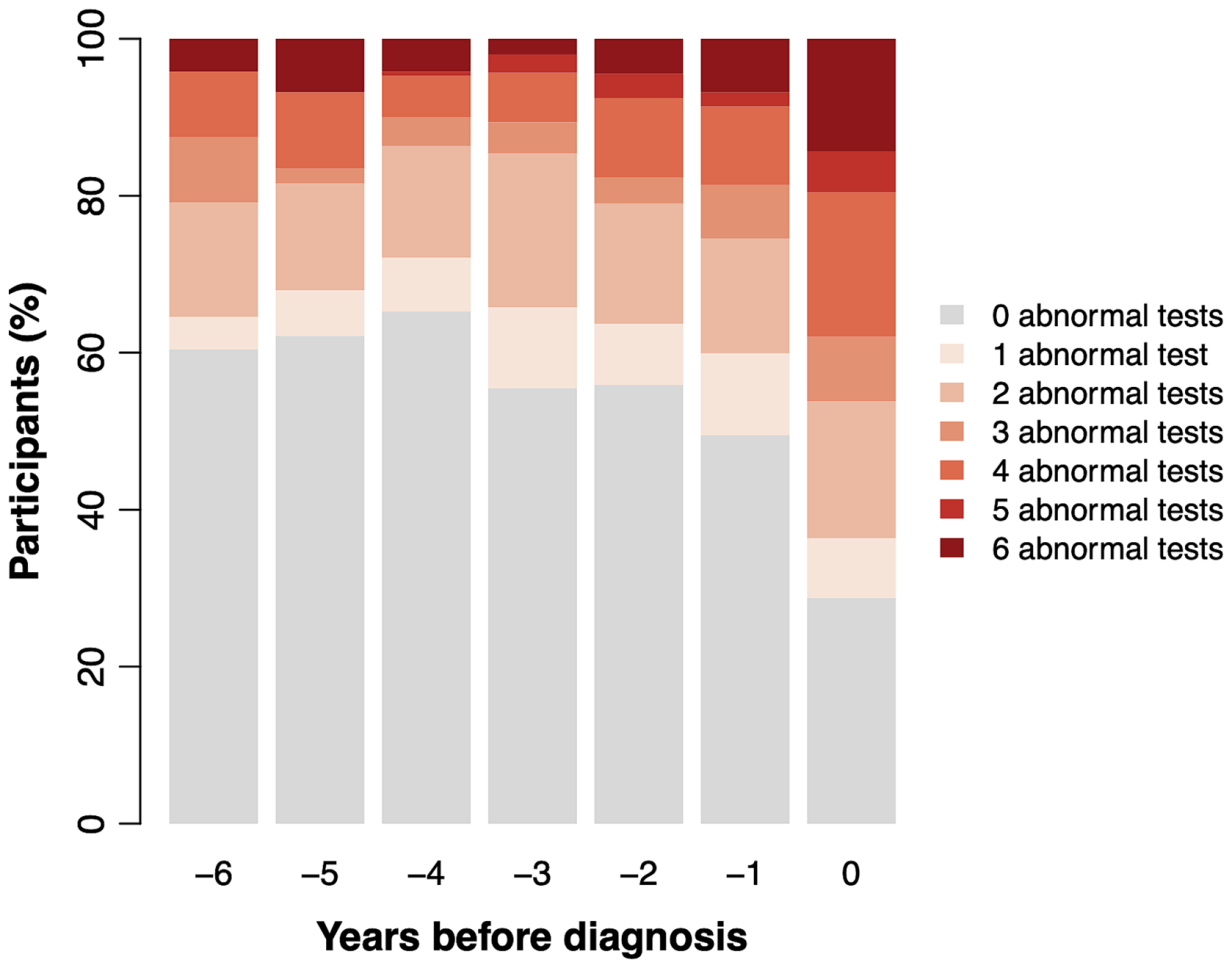
Abnormal oculomotor item counts preceding motor diagnosis in Enroll-HD data. The figure shows the distribution of abnormal oculomotor item counts, in the years leading up to manifest disease (DCL = 4), ranging from 0 to 6 abnormal tests in each individual. Only participants who transitioned to manifest Huntington’s disease are included and the number of included participants will be highest closest to diagnosis.

The slightly more irregular pattern of groupwise progression of saccade compared to pursuit items (Figure 1) may potentially reflect that these items are more challenging for patients to understand and perform consistently, and more examiner-dependent in terms of instructions and interpretation than smooth pursuit assessments. A digitalized assessment would probably help reduce examiner-related variability in saccade performance, but not necessarily the participant-related component. We note that the number of participants with available data was larger closer to the time of diagnosis, and the distribution of results could be expected to show some random variation across each premanifest year, as we are comparing non-identical groups (Figure 2).

As expected, all oculomotor variables were individually associated with time to diagnosis and all correlate with each other. Using stepwise logistic regression, we identified a model incorporating three different oculomotor items that were all independently significant, indicating that a combination of assessments provides more fine-grained prognostic information than a single item. The favored model included one smooth pursuit item, one vertical saccade item and one horizontal saccade item, thus capturing the breadth of abnormalities without including redundant variables. All three items remained individually significant when we validated the model in the independent PREDICT-HD dataset.

The recently proposed ISS-HD system replaces the traditional definition of “manifest HD” with staging based on the successive occurrence of biomarkers (stage 1), clinical signs (stage 2) and impairment of function (stage 3) (20). We anticipate that these milestones will have increasing importance in HD research as the ISS-HD is adopted in clinical trial design. The presence of definite oculomotor signs would indicate that a patient has reached stage 2, and within this stage, biomarkers that predict progression to stage 3 may be highly relevant for therapeutic trials. In stepwise regression analysis using stage 3 (TFC < 13) as event rather than DCL = 4 we found that the same three oculomotor items emerged as independently significant providing further support for this combination of assessments.

Our primary time-dependent Cox regression analysis treated oculomotor items as binary variables, either normal or abnormal. Only a very small proportion of participants had scores > 1 for any of the oculomotor items prior to diagnosis. Ideally, an optimized prediction model should account for the worse prognosis associated with scores of ≥ 2 compared to scores of 1, yet very rare observations will typically cause statistical challenges. Our attempts to model oculomotor variables as quantitative outcomes showed no linear increase in hazard with oculomotor scores ranging from 1 to 4, and no improved model fit compared to the simplified binary approach. This result illustrates the limitations in clinical assessments based on ordered categorical scores. Digital eye tracking biomarkers may generate truly scaled readouts, thus providing more differentiated output while also eliminating the statistical problem of rarely observed categories.

This study has several limitations. First, missing data across key variables, including oculomotor measures, led to reduced sample sizes in some analyses and may have introduced selection bias. Second, the timing of transition to manifest disease was based on visit assessments and therefore subject to interval censoring, as the exact time of transition between visits is unknown. Third, oculomotor measures were derived from clinician-rated UHDRS items, which may be subject to inter-rater variability and limited sensitivity to subtle changes. Ratings may be influenced by the evaluator’s expertise and training. Finally, although the large overall sample size is a strength, the number of premanifest participants with UHDRS oculomotor scores ≥2 was small, limiting statistical power for the models with non-binary coding of these variables.

In conclusion, we have studied the progression of oculomotor manifestations of HD in data from two large observational cohorts. We show that eye movements in the vertical plane are affected early in a larger proportion of individuals than horizontal, highlighting the importance of including vertical tests when developing digital eye tracking biomarkers. In our analyses, the full prognostic value of UHDRS oculomotor items was captured by the distinction between normal and abnormal tests, highlighting the limited resolution of subjective clinical ratings. We anticipate that digital eye movement biomarkers will become a valuable tool for therapeutic HD research, incorporating multiple oculomotor tasks and taking advantage of precise quantitative measurements to better capture subtle progression and detect treatment effects over time.

## Data Availability

Publicly available datasets were analyzed in this study. This data can be found at: https://www.enroll-hd.org/for-researchers/datasets/.
