# Supplementary material for: Assessing the prognostic value of early oculomotor abnormalities in Huntington’s disease
Source: Front Neurol. 2026 Jun 15;17:1844433. doi: 10.3389/fneur.2026.1844433 (PMC13310781; doi:10.3389/fneur.2026.1844433)
Supplement: Supplementary file 1 [file Data_Sheet_1.pdf]

Supplement to:

Assessing the prognostic value of early oculomotor abnormalities in Huntington's disease

Ahmad Kaddoura[1,2], Solveig E. J. Dalbro[1,2], Marleen R. van Walsem[1] and Lasse Pihlstrøm[1,2,*]

[1] Department of Neurology, Oslo University Hospital, Oslo, Norway

[2] Institute of Clinical Medicine, Faculty of Medicine, University of Oslo, Oslo, Norway

Supplementary Figure 1

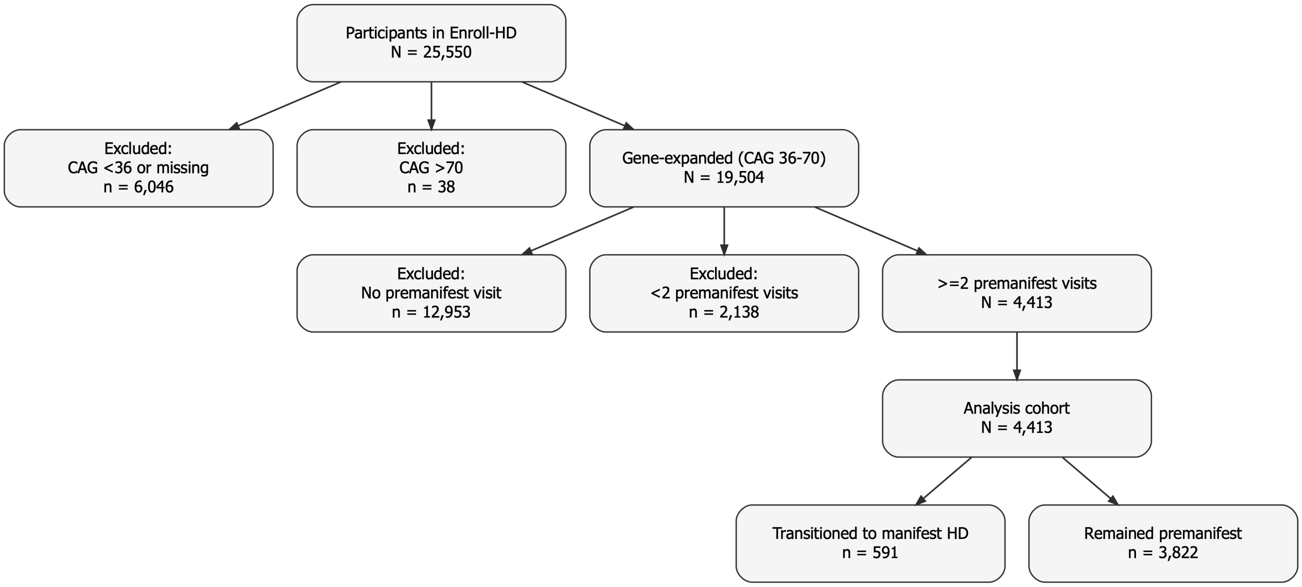

The figure illustrates the data selection process for the Cox proportional hazards regression analysis using clincial motor onset as event of interest in the Enroll-HD dataset.

Supplementary Table S1. Binary and quantitative time-dependent Cox regression analyses in the Enroll-HD TFC < 13 cohort.

	**Binary Cox models**		**Quantitative Cox models**	
**Oculomotor item**	**Single-item**	**Final model**	**Single-item**	**Final model**
Vertical smooth pursuit	1.63 (1.41-1.88) <0.001, PH = 0.081	1.20 (1.02-1.41) 0.027	1.50 (1.34-1.68) <0.001, PH = 0.073	1.16 (1.02-1.32), 0.025
Horizontal smooth pursuit	1.63 (1.40-1.90) <0.001, PH = 0.045	-	1.53 (1.36-1.73) <0.001, PH = 0.064	-
Vertical saccade velocity	2.00 (1.73-2.31) <0.001, PH = 0.300	1.41 (1.17-1.70) <0.001	1.60 (1.45-1.77) <0.001, PH = 0.113	-
Horizontal saccade velocity	2.06 (1.77-2.39) <0.001, PH = 0.440	-	1.77 (1.58-1.98) <0.001, PH = 0.289	1.43 (1.24-1.66), <0.001
Vertical saccade initiation	2.06 (1.78-2.39) <0.001, PH = 0.460	-	1.59 (1.44-1.76) <0.001, PH = 0.208	1.24 (1.08-1.42), 0.002
Horizontal saccade initiation	2.15 (1.86-2.50) <0.001, PH = 0.510	1.66 (1.38-1.99) <0.001	1.56 (1.41-1.71) <0.001, PH = 0.230	-
**Model performance**				
C-index (95% CI)	-	0.730 (0.713-0.748)	-	0.730 (0.713-0.748)
Bootstrap-corrected C-index	-	0.727	-	0.726
Person-years at risk	-	17,363	-	17,363
Global Schoenfeld p-value	-	0.34	-	0.26

Values are hazard ratios (HR) with 95% confidence intervals and p-values. PH indicates p-value from the Schoenfeld residual test of the proportional hazard’s assumption. Quantitative models treated oculomotor scores as ordinal 0–4 variables.

Supplementary Table S2. Comparison of coding strategies using AIC and BIC in Enroll-HD cohorts

Variable	Model	AIC	BIC
**Enroll-HD DCL4**			
Vertical saccade velocity	Binary (0 vs ≥1)	7400.3	7413.1
	Linear (0–4)	7415.4	7428.1
	Categorical (0 / 1 / ≥2)	7402.3	7419.3
Vertical smooth pursuit	Binary (0 vs ≥1)	7414.4	7427.2
	Linear (0–4)	7421.7	7434.4
	Categorical (0 / 1 / ≥2)	7416.1	7433.1
Horizontal saccade initiation	Binary (0 vs ≥1)	7417.9	7430.7
	Linear (0–4)	7425.2	7438.0
	Categorical (0 / 1 / ≥2)	7419.2	7436.2
**Enroll-HD TFC <13**			
Horizontal saccade velocity	Binary (0 vs ≥1)	12547.0	12561.1
	Linear (0–4)	12546.2	12560.5
	Categorical (0 / 1 / ≥2)	12544.8	12563.8
Vertical smooth pursuit	Binary (0 vs ≥1)	12592.0	12606.1
	Linear (0–4)	12587.3	12601.5
	Categorical (0 / 1 / ≥2)	12586.3	12605.3
Vertical saccade initiation	Binary (0 vs ≥1)	12544.4	12558.6
	Linear (0–4)	12560.0	12574.2
	Categorical (0 / 1 / ≥2)	12545.1	12564.1

Abbreviations: AIC, Akaike information criterion; BIC, Bayesian information criterion. Lower AIC and BIC values indicate better balance between model fit and model complexity. All models were adjusted for CAP score and sex. Binary models used dichotomized scores (0 vs ≥1), linear models treated UHDRS scores as ordinal 0–4 variables, and categorical models used grouped categories (0 / 1 / ≥2).

Supplementary Table S3. Exploratory hazard ratio analyses across ordered UHDRS severity categories.

Variable	1 vs 0 HR (p-value)	≥2 vs 0 HR (p-value)	≥2 vs 1 HR (p-value)
**Enroll-HD DCL4**			
Vertical saccade velocity	1.72 (<0.001)	1.14 (0.710)	0.66 (0.220)
Vertical smooth pursuit	1.54 (<0.001)	0.74 (0.420)	0.48 (0.045)
Horizontal saccade initiation	1.55 (<0.001)	1.08 (0.750)	0.70 (0.160)
**Enroll-HD TFC <13**			
Horizontal saccade velocity	1.59 (<0.001)	1.81 (0.001)	1.14 (0.420)
Vertical smooth pursuit	1.25 (0.008)	1.29 (0.140)	1.04 (0.830)
Vertical saccade initiation	1.56 (<0.001)	1.46 (0.020)	0.94 (0.640)

Abbreviations: HR, hazard ratio. Exploratory HR analyses were used to assess risk patterns across ordered UHDRS severity categories (0, 1, and ≥2). Results were used for interpretation of risk progression patterns rather than formal model comparison.

Supplementary Table S4. Quantitative time-dependent Cox regression analyses in the DCL = 4 cohorts.

Oculomotor item	Enroll-HD Single-item	Enroll-HD Final model	PREDICT-HD Final model
Vertical smooth pursuit	1.57 (1.35-1.83) <0.001, PH = 0.914	1.25 (1.04-1.51), 0.020	1.34 (1.04–1.72), 0.022
Horizontal smooth pursuit	1.56 (1.30-1.86) <0.001, PH = 0.986	-	-
Vertical saccade velocity	1.59 (1.38-1.83) <0.001, PH = 0.962	1.29 (1.07-1.56), 0.009	1.49 (1.15-1.92), 0.002
Horizontal saccade velocity	1.66 (1.41-1.96) <0.001, PH = 0.991	-	-
Vertical saccade initiation	1.55 (1.33-1.80) <0.001, PH = 0.996	-	-
Horizontal saccade initiation	1.50 (1.29-1.73) <0.001, PH = 0.997	1.21 (1.02-1.44), 0.031	1.61 (1.32-1.97), <0.001
Model performance			
C-index (95% CI)		0.747 (0.727–0.767)	0.806 (0.773-0.838)
Bootstrap-corrected C-index		0.745	0.805
Person-years at risk		16,751	4,847
Global Schoenfeld p-value		0.98	0.17

Values are hazard ratios (HR) with 95% confidence intervals and p-values. PH indicates p-value from the Schoenfeld residual test of the proportional hazard's assumption. Quantitative models treated oculomotor scores as a linear 0–4 variable.
